# Cannabis use and violent behavior

**DOI:** 10.1192/j.eurpsy.2022.2159

**Published:** 2022-09-01

**Authors:** M. Roque Gonçalves, A. Elias De Sousa, A.S. Machado, A. Silva, M. Vieira-Coelho

**Affiliations:** 1 Centro Hospitalar Universitário de São João, Serviço De Psiquiatria, Porto, Portugal; 2 FMUP, Departamento De Neurociências Clínicas E Saúde Mental, Porto, Portugal; 3 FMUP, Biomedicina, Porto, Portugal

**Keywords:** Cannabis, THC, young-adults, violence

## Abstract

**Introduction:**

Cannabis is the most commonly used psychoactive drug, particularly among adolescents and young adults. Accordingly, to the European Drug Report 2021, the prevalence of cannabis use is about five times that of other substances, so that last year cannabis use among EU inhabitants aged 15 to 24y is estimated at 19.2 %. Even though most human research has concluded that Δ9-tetrahydrocannabinol (THC), tends to dampen rather than provoke aggression in acute doses, recent evidence suggests a relationship between cannabis usage and violent behavior, especially when associated with neurodevelopment stages.

**Objectives:**

To review the existing evidence on the association between cannabis and violence in young adults and provide an overview of possible mechanisms explaining this relation.

**Methods:**

Literature review was based on PubMed/ MEDLINE, using key words inclusive for violence, cannabis and adolescence. Studies included focused the young-adults population and considered the relation between cannabis use and behaviors reported as acts of physical violence. Studies were excluded if they included self-harm behaviors.

**Results:**

Recent studies, including case-reports, showed a global moderate association between cannabis use and violence. Preliminary data has even highlighted a potential larger effect in more frequent users. Also, the cannabis role in the central nervous system (CNS), with most expression in the limbic cortices, and especially as it participates in a variety of brain function modulations - including executive functions, inhibition/impulsivity, and emotional control, has been pointed as one of the main arguments for this relation.

**Conclusions:**

Further studies may shed light on the effects of cannabis use on behavior.

**Disclosure:**

No significant relationships.

